# Association between urinary arsenic, blood cadmium, blood lead, and blood mercury levels and serum prostate-specific antigen in a population-based cohort of men in the United States

**DOI:** 10.1371/journal.pone.0250744

**Published:** 2021-04-23

**Authors:** Hongke Wu, Ming Wang, Jay D. Raman, Alicia C. McDonald

**Affiliations:** 1 Department of Public Health Sciences, Pennsylvania State University College of Medicine, Hershey, Pennsylvania, United States of America; 2 Penn State Cancer Institute, Hershey, Pennsylvania, United States of America; 3 Department of Surgery, Pennsylvania State Milton S. Hershey Medical Center, Hershey, Pennsylvania, United States of America; Graduate School of Public Health and Health Policy, City University of New York, UNITED STATES

## Abstract

Exposures to heavy metals have been linked to prostate cancer risk. The relationship of these exposures with serum prostate-specific antigen (PSA), a marker used for prostate cancer screening, is unknown. We examined whether total urinary arsenic, urinary dimethylarsonic acid, blood cadmium, blood lead, and total blood mercury levels are associated with elevated PSA among presumably healthy U.S. men. Prostate cancer-free men, aged ≥40 years, were identified from the 2003–2010 National Health and Nutrition Examination Survey. Logistic regression analyses with survey sample weights were used to examine the association between heavy metal levels and elevated PSA for the total population and stratified by black and white race, after adjusting for confounders. There were 5,477 men included. Approximately 7% had elevated PSA. Men with an elevated PSA had statistically significantly higher levels of blood cadmium and blood lead compared to men with a normal PSA (p-values ≤ 0.02), with black men having higher levels. After adjusting for age, race/ethnicity, body mass index, smoking, and education, there was no association found between any of the heavy metal levels and elevated PSA for the total population. In addition, there was no association found when stratified by black and white race. Further investigation is warranted in a larger cohort of men who persistently are exposed to these heavy metals.

## Introduction

Black race, older age, and family history of prostate cancer are well-established risk factors for prostate cancer, the most common cancer among U.S. men [1]. However, there are several risk factors for prostate cancer that are less clear such as environmental exposures to heavy metals. Human and animal studies suggest that exposures to heavy metals may play a role in carcinogenesis [2–7]. Toxic exposures to heavy metals such as arsenic (As) and cadmium (Cd), either through inhalation and/or ingestion, have been linked to various cancers including prostate cancer risk and mortality [3,7–13]. Other heavy metals such as lead (Pb) and mercury (Hg) have been less clear with their relationship with prostate cancer due to limited studies [2,14]. The International Agency for Research on Cancer has classified As and Cd as carcinogens to humans (Group 1) [7] and Pb and Hg (i.e., methylmercury compound) as probable (Group 2A) and possible (Group 2B) carcinogens to humans [2,14], respectively. However, study findings of the relationship between these heavy metals and prostate cancer have been mixed [3,7,15,16]; therefore, their role in prostate carcinogenesis remains unclear. In addition, these studies were conducted in a majority white male population; therefore, relationships between these heavy metal exposures and prostate cancer by race, in particular, among black men who are at higher risk for prostate cancer remain not known and therefore warrant further investigation.

Chronic Inflammation has been identified as a probable risk factor for prostate cancer through its initiation of cancer cellular processes that lead to cellular and/or genome damage and/or increased oxidative stress [17–19]. As and Cd exposures are known to influence cancer processes such as cellular proliferation and differentiation, apoptosis, and/or angiogenesis as well as inhibit DNA repair and induce oxidative stress in cell-based models [4–7,20]. Pb has been suggested to influence cancer processes through cell toxicity and gene mutation [2] and for Hg (i.e., methylmercury compound) through chromosomal aberration [14]. The relationship between these heavy metals and prostatic inflammation, a probable risk factor for prostate cancer, is not clear.

Serum prostate-specific antigen (PSA), a serine protease that is produced by normal and malignant epithelial cells in the prostate, is not only a marker used for prostate cancer screening, but also, it identifies men of various prostatic conditions such a benign prostatic hyperplasia (BPH). Therefore, PSA is prostate-specific and not prostate cancer-specific. However, men with elevated serum PSA levels determined by age and race are considered at higher risk for prostate cancer. In addition, acute and chronic prostatic inflammation has been shown to be associated with elevated serum PSA levels [21]. However, few studies have examined relationships between heavy metal exposures, possible contributors to chronic inflammation, and serum PSA. We conducted a cross-sectional study to determine whether levels of total urinary As, urinary dimethylarsonic acid (DMA) (a speciated As), blood Cd, blood Pb, and total blood Hg are associated with elevated PSA among U.S men who are cancer-free for the total population and stratified by black and white race. Investigating relationships between these heavy metals and elevated PSA, a marker associated with benign and malignant prostatic diseases, may provide insight on possible influences these heavy metals may have on prostatic inflammation and prostate cancer risk.

## Materials and methods

### Study population

The study population consisted of men, aged ≥ 40 years, who participated in the population-based National Health and Nutrition Examination Survey (NHANES), a nationally representative survey of the U.S. non-institutionalized population, from 2003 to 2010, years in which both data on serum PSA and heavy metals were available. Demographic information (age, race/ethnicity, and education), medical history (i.e, prostate cancer diagnosis), family history of prostate cancer, cigarette smoking, body mass index (BMI), and laboratory measurements (serum PSA, total urinary As, urinary DMA, urinary arsenobetaine, blood Cd, blood Pb, total blood Hg, and urinary creatinine) were extracted from the NHANES datasets. Men were included if they had a serum PSA result, did not have a cancer diagnosis, and had a least one heavy metal result. Of the 7,140 men ages ≥ 40 years who participated in the 2003–2010 NHANES, there were 6,018 men who had a serum PSA result. Of these men, 541 were excluded due to a prostate cancer or other cancer diagnosis (N = 538) or who had a missing heavy metal result (N = 3). There were 5,477 men included in the total population analyses (S1 Fig.). Among these men, total urinary As and urinary DMA results were available for 1,812 of them; and, blood Cd, blood Pb, and blood Hg results were available for 5,474 of them. There were 3 men who had both available total urinary As and urinary DMA results but did not have available blood Cd, blood Pb, and blood Hg results.

The limit of detection (LOD) for total urinary As and urinary DMA was 0.83% and 12.1%, respectively [22]. Urinary arsenobetaine (LOD = 27.6%) was included in the sub-analysis to account for the influence of non-toxic seafood have on toxic As [23]. For total urinary As, urinary DMA, and urinary arsenobetaine, levels that were below LOD were divided by the square-root of two. For blood Cd, blood Pb, and total blood Hg, levels that were below the LOD were assigned a constant value in the NHANES database [24].

Serum PSA levels were categorized in the analyses as normal and elevated levels based on white and black races’ age-specific PSA cutoffs for a prostate biopsy [25]. Elevated serum PSA levels were defined as the following for white and black races by age group: 1) white men (40–49 years: > 2.5 ng/mL; 50–59 years: > 3.5 ng/mL; 60–69 years: > 4.5 ng/mL; and 70–79 years: > 6.5 ng/mL) and 2) black men (40–49 years: > 2.0 ng/mL; 50–59 years: > 4.0 ng/mL; 60–69 years: > 4.5 ng/mL; and 70–79 years: > 5.5 ng/mL) [25]. Because race-specific PSA was not listed for all racial/ethnic groups (i.e., Hispanic), white race’s age-specific PSA cutoffs were applied to the other races and Hispanic ethnicity.

To participate in NHANES, informed consent was obtained from all study participants. This study was conducted in accordance with the Declaration of Helsinki. All data were approved by the National Center for Health Statistics Research Ethics Review Board.

### Laboratory analysis

Total urinary As levels were determined by inductively Coupled Plasma Dynamic Reaction Cell Mass Spectrometry (ICP-DRC-MS) [22]. Speciated urinary As levels (i.e., DMA and arsenobetaine) were determined by using high performance liquid chromatography coupled to an ICP-DRC-MS [22]. Whole blood Pb, Cd, and total Hg levels were determined using inductively coupled plasma mass spectrometry [26]. Total PSA values were measured using the Access Hybritech free PSA assay [27].

### Data analysis

Descriptive analyses of study variables were presented by median values with their range or 25–75 percentile for continuous variables and frequencies with proportions for categorical variables. To compare differences between men with normal and elevated serum PSA groups, Pearson’s *Χ*^2^ test was used for categorical variables; and, Wilcoxon rank sum test was used for continuous variables. Spearman correlation coefficient (r) was calculated to examine the correlation between each of the heavy metal levels and serum PSA. The normality of all heavy metal and creatinine levels was evaluated; these levels were natural log-transformed due to the lack of normality. Geometric mean (GM) and its 95% confidence interval (CI) were calculated for each heavy metal level. Logistic regression with sampling weights was used to compare heavy metals’ GM between serum PSA groups in which the p-values were calculated based on t-tests for the total population, between black and white men, and within black and white men.

Age-adjusted and multivariable logistic regression models with survey sample weights were used to determine the association between each heavy metal level and elevated PSA by calculating the odds ratio (OR) and its 95% CI for the total study population and in black and white men, separately. The following covariates were included in the logistic regression models to adjust for potential confounders of serum PSA: 1) continuous age (Model 1) and 2) continuous age, race/ethnicity, continuous BMI, smoking, and education (Model 2). For the total urinary As and urinary DMA analyses, urinary creatinine was included in all logistic regression models. In sub-analyses, urinary levels of total As and DMA were regressed on arsenobetaine to account for the influence nontoxic seafood As have on total As and DMA at low levels of exposure; details of this methodology were described elsewhere [23]. The interaction term between race and each heavy metal level was included in each logistic regression model and was excluded in the model if it was not statistically significant. Regardless of whether an interaction was observed, logistic regression models were stratified by black and white men in order to investigate whether there were differences between associations between the heavy metal levels and elevated serum PSA within each race.

All hypothesis tests were two-sided and conducted at the significance level of 0.05 in SAS 9.4 (Cary, NC).

## Results

There was a total of 5,477 men who had met the study criteria. The majority of the study population were of white race (50.79%; N = 2782/5477). There were 6.99% (N = 383/5,477) of the men who had an elevated PSA. There were 10.48% (N = 112/1069) among black men and 5.75% (N = 160/2782) among white men who had an elevated PSA. Overall, men with an elevated PSA were more likely to be older in age, have a lower education level, and have a lower BMI compared to men with a normal PSA (p-values ≤ 0.01) (Table 1).

**Table 1:** Description of 2003–2010 NHANES Men by serum PSA levels.

Study Variables	Normal PSA	Elevated PSA	*P*-value
**Age (in years)**	N = 5094	N = 383	
Median (range)	57 (40–85)	66 (40–85)	< .0001
**Body mass index (kg/m**^**2**^**)**	N = 5009	N = 372	
Median (25–75 percentile)	28.23 (25.30–31.67)	27.23 (24.95–30.21)	< .0001
**Race/Ethnicity N (%)**	N = 5094	N = 383	
Non-Hispanic White	2622 (51.47)	160 (41.78)	< .0001
Non-Hispanic Black	957 (18.79)	112 (29.24)	
Hispanic	1307 (25.66)	96 (25.07)	
Others	208 (4.08)	15 (3.92)	
**Education N (%)**	N = 5088	N = 382	
≤ 12 years (Did not complete high school)	1606 (31.56)	145 (37.96)	0.01
High school graduate or greater	3482 (68.44)	237 (62.04)	
Family history of prostate cancer N (%)*	N = 3471	N = 265	
No	3013 (86.80)	224 (84.53)	0.29
Yes	458 (13.20)	41 (15.47)	
**Smoked at least 100 cigarettes in life N (%)**	N = 5090	N = 383	
No	1948 (38.27)	155 (40.47)	0.39
Yes	3142 (61.73)	228 (59.53)	

Note

*This study variable was not available in the 2009–2010 NHANES; Pearson’s X^2^ test was used for categorical variables; Wilcoxon rank sum test was used for continuous variables; PSA = prostate-specific antigen; and, NHANES = National Health and Nutrition Examination Survey.

For the total population, men with an elevated PSA had statistically significantly higher levels of blood Cd and blood Pb compared to men with a normal PSA (p-values ≤ 0.02) (Table 2). When stratified by black and white race, there were statistically significantly higher levels of blood Pb for both black and white men with an elevated PSA compared to black and white men with a normal PSA, respectively (p-values < 0.01; Data not shown). Black men had statistically significantly higher levels for all of the heavy metals compared to white men, overall and among men with a normal PSA (p-values < 0.01; Data not shown). Among men with an elevated PSA, black men had statistically significantly higher levels of blood Cd, blood Pb, and blood Hg compared to white men (p-value ≤ 0.05; Data not shown). The correlation coefficient (r) was < 0.4 between each of the heavy metal levels and serum PSA, independently (Data not shown); therefore, there was no moderate or strong correlation observed between any of the heavy metal level and serum PSA.

**Table 2:** Geometric mean of heavy metals by serum PSA levels among 2003–2010 NHANES Men.

Heavy Metals GM (95% CI; N)	Normal PSA	Elevated PSA	*P-value*
Urinary arsenic, total (ug/L)	11.000 (10.412,11.622; 1683)	11.667 (9.556, 14.244; 129)	0.573
Urinary dimethylarsonic acid (ug/L)	4.301 (4.138, 4.471; 1670)	4.412 (3.828, 5.087; 130)	0.732
Blood cadmium (ug/L)	0.397 (0.388, 0.405; 5091)	0.435 (0.404, 0.469; 383)	0.019
Blood lead (ug/dL)	2.066 (2.033, 2.099; 5091)	2.411 (2.269, 2.561; 383)	< .001
Blood mercury, total (ug/L)	1.044 (1.016, 1.073; 5091)	1.081 (0.975, 1.198; 383)	0.525

Note: Logistic regression was used to compare geometric means between serum PSA groups in which the p-values were calculated based on t-tests; PSA = prostate-specific antigen; NHANES = National Health and Nutrition Examination Survey; GM = geometric mean; and, CI = confidence interval.

For the total population and in black and white men, separately, there was no association between each of the heavy metal levels and elevated PSA in the age-adjusted and multivariate analyses (Tables 3 and 4). For the sub-analyses where seafood was accounted for total urinary As and urinary DMA, no association was observed with elevated serum PSA in the age-adjusted and multivariate analysis for the total population and among black and white men, separately (S1 Table).

**Table 3:** Associations between urinary levels of total arsenic and dimethylarsonic acid and elevated serum PSA among 2003–2010 NHANES Men.

	Model 1		Model 2	
Heavy Metals	# Elevated / Normal / PSA	OR (95% CI)	# Elevated / Normal PSA	OR (95% CI)
**Total Population**				
Urinary arsenic, total (ug/L)	129/1683	1.018 (0.774, 1.339)	125/1663	0.911 (0.674, 1.232)
Urinary dimethylarsonic acid (ug/L)	130/1670	1.193 (0.818, 1.740)	126/1650	0.971 (0.637, 1.482)
**Black Men**				
Urinary arsenic, total (ug/L)	38/318	1.109 (0.859, 1.432)	36/313	1.033 (0.775, 1.376)
Urinary dimethylarsonic acid (ug/L)	38/317	0.870 (0.567, 1.335)	36/312	0.781 (0.482, 1.265)
**White Men**				
Urinary arsenic, total (ug/L)	47/866	0.866 (0.571, 1.374)	47/856	0.857 (0.549, 1.340)
Urinary dimethylarsonic acid (ug/L)	49/856	1.162 (0.692, 1.953)	49/846	1.040 (0.606, 1.787)

Note: Model 1 logistic regression with sampling weights was adjusted for continuous age and creatinine; Model 2 logistic regression with sampling weights was adjusted for continuous age, race/ethnicity (total population only), continuous body mass index, cigarette smoking, education, and creatinine; PSA = prostate-specific antigen; NHANES = National Health and Nutrition Examination Survey; OR = odds ratio; CI = confidence interval; and, OR was calculated as one-unit increase for each heavy metal level.

**Table 4:** Associations between blood levels of cadmium, lead, and total mercury and elevated serum PSA among 2003–2010 NHANES Men.

	Model 1		Model 2	
Heavy Metals	# Elevated / Normal / PSA	OR (95% CI)	# Elevated / Normal PSA	OR (95% CI)
**Total Population**				
Blood cadmium (ug/L)	383/5091	1.010 (0.849, 1.202)	3714998	1.022 (0.842, 1.241)
Blood lead (ug/dL)	383/5091	1.223 (0.966, 1.549)	371/4998	1.129 (0.878, 1.451)
Blood mercury, total (ug/L)	383/5091	1.070 (0.910, 1.259)	371/4998	1.039 (0.870, 1.243)
**Black Men**				
Blood cadmium (ug/L)	112/957	1.189 (0.900, 1.570)	107/933	1.261 (0.912, 1.744)
Blood lead (ug/dL)	112/957	1.293 (0.957, 1.747)	107/933	1.234 (0.876, 1.737)
Blood mercury, total (ug/L)	112/957	1.017 (0.783, 1.321)	107/933	0.995 (0.760, 1.302)
**White Men**				
Blood cadmium (ug/L)	160/2619	0.913 (0.715, 1.166)	156/2565	0.982 (0.762, 1.266)
Blood lead (ug/dL)	160/2619	1.254 (0.915, 1.719)	156/2565	1.288 (0.937, 1.771)
Blood mercury, total (ug/L)	160/2619	0.973 (0.806, 1.176)	156/2565	0.951 (0.769, 1.176)

Note: Model 1 logistic regression with sampling weights was adjusted for continuous age; Model 2 logistic regression with sampling weights was adjusted for continuous age, race/ethnicity (total population only), continuous body mass index, cigarette smoking, and education; PSA = prostate-specific antigen; NHANES = National Health and Nutrition Examination Survey; OR = odds ratio; CI = confidence interval; and, OR was calculated as one-unit increase for each heavy metal level.

## Discussion

To our knowledge, this is the first study to investigate the relationship between the levels of total urinary As, urinary DMA, blood Cd, blood Pb, and total blood Hg and elevated serum PSA defined by age and race in a presumably healthy cohort of U.S. men. There were higher levels of urinary Cd and blood Pb in men with elevated serum PSA compared to men with normal serum PSA, with black men having higher levels compared to white men. However, after adjusting for age, race/ethnicity, BMI, cigarette smoking, and education, these heavy metals were not associated with elevated serum PSA for the total population and among black and white men, separately. Possible reasons for these study findings could be due to low exposures to these heavy metals in the U.S. general population or no true relationship exists between these heavy metals and serum PSA.

Few studies have examined relationships between Cd exposures and serum PSA; and, to our knowledge, relationships between Pb and Hg and serum PSA have not been examined. For Cd studies, a study conducted in China found a dose-response relationship between Cd exposure and serum PSA among prostate cancer cases with an abnormal PSA [28]. Another study conducted in the U.S. general population found a positive correlation between urinary Cd levels and PSA levels among men who had an zinc intake < 12.7 mg/day; but, no association was found between urinary Cd levels and elevated PSA among these men [29]. This study finding of no association between urinary Cd levels and elevated PSA is consistent with the present study finding of no association between blood Cd levels and elevated PSA, after adjusting for potential confounders. In the general population, exposure to Cd is mostly in one’s diet among non-smokers and cigarette smoke among smokers [30]. A possible reason for the no association between blood Cd levels and elevated PSA in the present study could be due to low exposures to Cd, either through diet or inhalation, in this study population compared to populations where exposure to this heavy metal may be prevalent such as occupational populations. Another possible reason for no association could be that exposures to Cd do not influence serum PSA levels. As for Pb and Hg, more studies are needed to evaluate their relationships with serum PSA.

Total urinary As and urinary DMA were found not to be associated with elevated serum PSA, even after accounting for urinary arsenobetaine which is typically used as a measure of overall seafood exposure [23]. The present study findings are consistent with a previous study that found no difference in total serum PSA between men exposed compared to men not exposed to high concentrations of As in drinking water [31]. However, another study found a statistically significant positive correlation between urinary As levels and serum PSA among copper foundry workers [32]. This no association with these heavy metals and elevated PSA may be due to the low levels found in this study population compared to occupational populations where these exposures are prevalent. Further studies are warranted in study population where exposures to As and DMA are prevalent to examine their possible relationship with serum PSA.

Black men are known to have a higher prostate cancer risk and more aggressive prostate cancer compared to other racial groups [1,33,34]. They are also known to have higher PSA levels compared to other racial groups [35,36]. Studies have shown African-Americans, in particular women and children, having higher blood levels of Pb, Cd, and Hg compared to their white counterparts [37–40]. To our knowledge, no study has reported relationships between heavy metal exposures and serum PSA among black men. In the present study, there was a higher proportion of black men having elevated serum PSA levels and higher heavy metal levels compared to white men; however, there was no association found between these heavy metals and elevated PSA among black men, after adjusting for potential confounders. Possible reasons for the lack of association could be due to low exposures to these heavy metals in this study population or no true association exists between these heavy metals and elevated PSA among these men.

A major strength of the present study is that it is a population-based study that examined whether there are black and white racial differences in the association between the levels of heavy metals and elevated PSA among a presumably healthy cohort of men. However, there were some limitations to this study. One limitation is the cross-sectional nature of the survey which lacks follow-up information such as prostate cancer outcome and whether heavy metal exposures preceded elevated PSA levels among these men. Therefore, relationships between these heavy metal exposures and prostate cancer and elevated PSA risk were not examined. A second limitation is that the NHANES study data were limited to years 2003 to 2010 due to the availability of serum PSA and heavy metal data, and therefore, more recent NHANES study data were not used. Exposures to these heavy metals may have changed over time which may provide a different study findings than the ones found in the present study. A third limitation was that there were missing data for some men (i.e., family history of prostate cancer and urinary total and speciated As levels), resulting in a smaller sample size for some analyses which may have reduced the study’s power and possibly affect the study association found. A fourth limitation was the potential of information bias such as inaccurate information on family history of prostate cancer that was assessed through a questionnaire which can affect the association observed. A fifth limitation is that NHANES did not specify other racial groups such as the Asian and American Indian races who were reported in previous studies to have higher biological levels of As and several heavy metals, respectively, compared to U.S. blacks and whites [41,42]. As a result, we were unable to look at the association between heavy metal levels and elevated PSA within other racial groups. A sixth limitation is that there was a small number of individuals with heavy metal levels that were below the LOD; these levels were imputed by a constant value which may lead to a biased estimation. Finally, prostatic conditions such as BPH that may influence serum PSA levels were not evaluated and adjusted for in the analyses due to missing data on the majority of the men (89.3%); therefore, there is the chance that the study findings may not be a true association between these heavy metal levels and elevated PSA.

## Conclusions

In conclusion, higher levels of blood Cd and blood Pb were found among men with elevated serum PSA compared to men with normal PSA. However, there was no association found between any of these heavy metal levels and elevated PSA for the total population and among black and white men, separately, after adjusting for potential confounders. Investigating relationships between heavy metal exposures and serum PSA in a larger population where these exposures are prevalent, either inhalation and/or ingestion, may provide better insight on their possible influence on serum PSA, a marker associated with prostate cancer risk and prostatic inflammation.

## Supporting information

S1 Fig Flowchart of eligible men, aged ≥ 40 years, from 2003–2010 NHANES. (TIF)

S1 Table Associations between urinary levels of total arsenic and dimethylarsonic acid and elevated Serum PSA among NHANES Men, after removing the effects of seafood. (DOCX)
