# Supplementary figures and images for: Association between urinary arsenic, blood cadmium, blood lead, and blood mercury levels and serum prostate-specific antigen in a population-based cohort of men in the United States
Source: PLoS One. 2021 Apr 23;16(4):e0250744. doi: 10.1371/journal.pone.0250744 (PMC8064543; doi:10.1371/journal.pone.0250744)

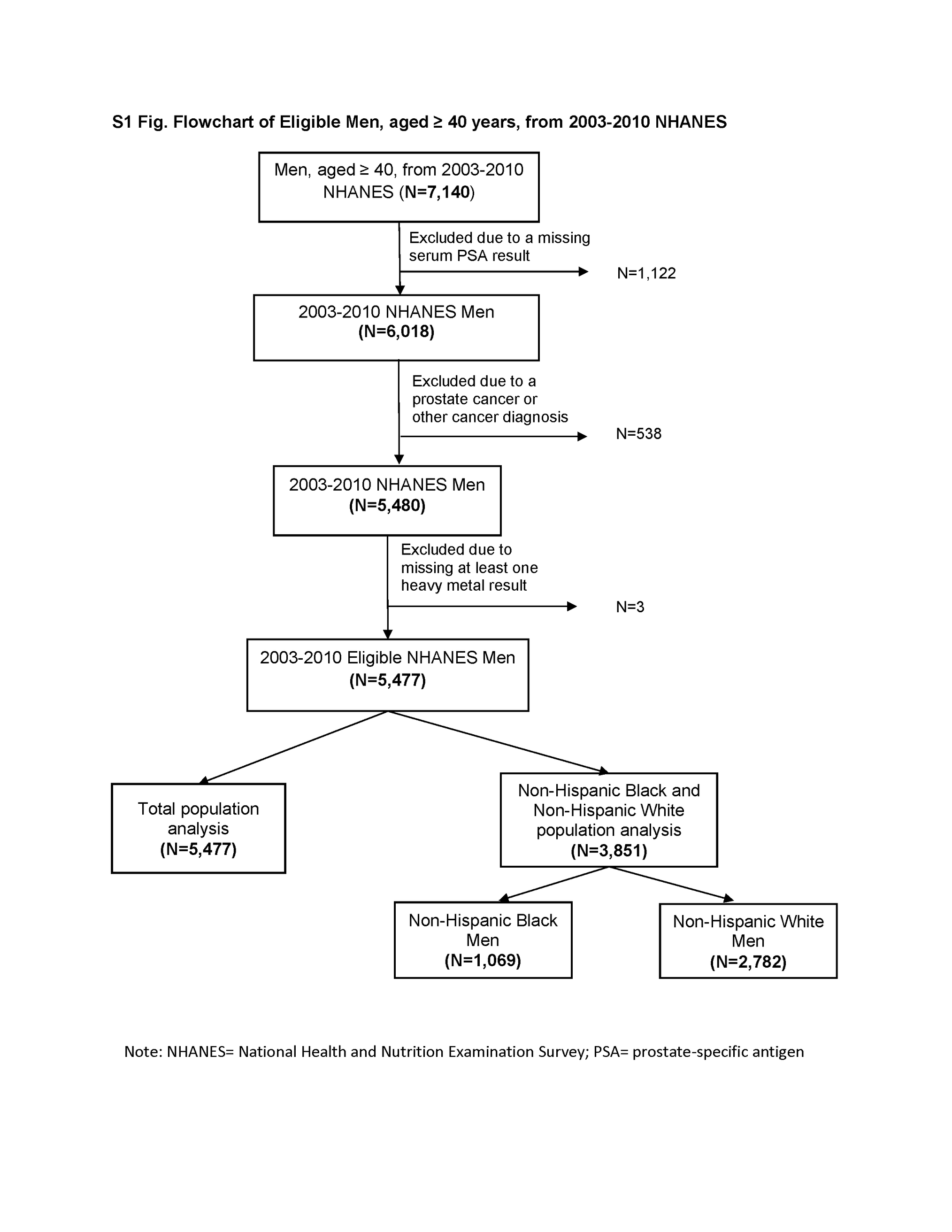

Supplement: S1 Fig — (TIF) [file pone.0250744.s001.tif]
