# Supplementary material for: Association between urinary arsenic, blood cadmium, blood lead, and blood mercury levels and serum prostate-specific antigen in a population-based cohort of men in the United States
Source: PLoS One. 2021 Apr 23;16(4):e0250744. doi: 10.1371/journal.pone.0250744 (PMC8064543; doi:10.1371/journal.pone.0250744)
Supplement: S1 Table — (DOCX) [file pone.0250744.s002.docx]

**S1 Table. Associations between Urinary Levels of Total Arsenic and Dimethylarsonic Acid and Elevated Serum PSA among NHANES Men, after Removing the Effects of Seafood**

|  | **Model 1** |  | **Model 2** |  |
| --- | --- | --- | --- | --- |
| **Heavy Metals** | **# Elevated / Normal PSA** | **OR (95% CI)** | **# Elevated / Normal PSA** | **OR (95% CI)** |
| **Total Population** | | | | |
| Urinary arsenic, total (ug/L) | 128/1664 | 0.912 (0.487, 1.709) | 124/1645 | 0.795 (0.391, 1.615) |
| Urinary dimethylarsonic acid (ug/L) | 130/1670 | 1.192 (0.785, 1.809) | 126/1650 | 0.995 (0.603, 1.642) |
| **Black Men** | | | | |
| Urinary arsenic, total (ug/L) | 38/317 | 0.900 (0.447, 1.813) | 36/312 | 0.878 (0.455, 1.733) |
| Urinary dimethylarsonic (ug/L) | 38/317 | 0.703 (0.392, 1.261) | 36/312 | 0.658 (0.362, 1.199) |
| **White Men** | | | | |
| Urinary arsenic, total (ug/L) | 47/852 | 0.735 (0.261, 2.065) | 47/843 | 0.679 (0.237, 1.942) |
| Urinary dimethylarsonic acid (ug/L) | 49/856 | 1.225 (0.662, 2.266) | 49/846 | 1.092 (0.560, 2.129) |

Note: Model 1 logistic regression with sampling weights was adjusted for continuous age and creatinine; Model 2 logistic regression with sampling weights was adjusted for continuous age, race (total population only), continuous body mass index, cigarette smoking, education, and creatinine; PSA= prostate-specific antigen; NHANES= National Health and Nutrition Examination Survey; OR= odds ratio; CI= confidence interval; and, OR was calculated as one-unit increase for each heavy metal level.
